# Intraneural Posterior Interosseous Nerve Lipoma with Complete Paralysis: Case Report and Review of the Literature

**DOI:** 10.7759/cureus.2689

**Published:** 2018-05-25

**Authors:** Achal P Patel, Salah G Aoun, Mazin Al Tamimi

**Affiliations:** 1 Division of Neurosurgery, University of Texas Medical Branch at Galveston; 2 Neurological Surgery, University of Texas Southwestern Medical Center, Dallas, USA

**Keywords:** intraneural, lipoma, posterior interosseous nerve

## Abstract

An intraneural posterior interosseous nerve (PIN) lipoma is a rare entity consisting of two types and only two previously reported cases. Treatment involves total excision for the well-encapsulated “true intraneural lipomas” type and subtotal resection for the other type, fibrolipomatous hamartomas of the nerve. We present the management and surgical treatment of a case that illustrates a variation of the traditional posterolateral surgical approach for the complete excision of an intraneural PIN lipoma—contrary to the more commonly used anterior approach in literature—along with a literature review of intraneural PIN lipomas.

## Introduction

Posterior interosseous nerve (PIN) lesions are infrequent and, when found, often originate from the nerve sheath. However, other soft tissue lesions, such as lipomas, can also cause neural compression symptoms. Most of these lesions originate from nearby musculoskeletal structures, but few case reports of intraneural lipomas have been reported. We describe a case report of an intraneural PIN lipoma and review the previously reported cases in the literature along with its clinical evaluation and surgical management.

## Case presentation

**Figure 1:**
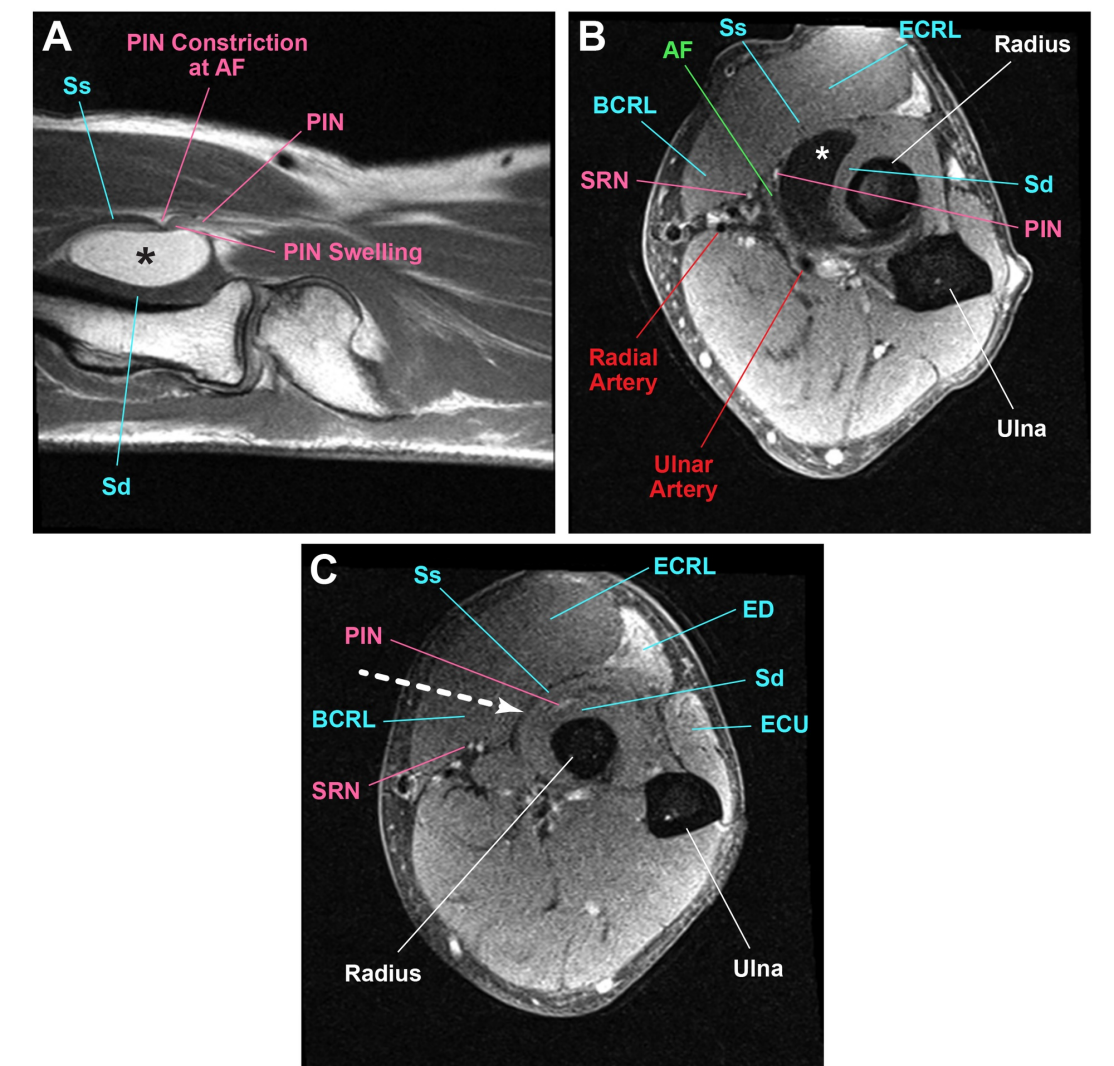
MRI showing a homogeneously hyperintense lesion (A) T1-weighted sagittal magnetic resonance imaging shows a hyperintense lipoma lesion (*) causing the swelling of the PIN proximal to the constriction at the AF. (B) T2-weighted axial shows the PIN entering between the AF and the lipoma. (C) T2-weighted axial further distally shows the PIN continuing distally between the superficial (Ss) and deep (Sd) heads of the supinator. The direction of approach between the BCRL and ECRL is shown (white arrow). Hyperintensity is seen in the ED and ECU. Abbreviations (a-z): Arcade of Frohse (AF), brachioradialis (BCRL), extensor carpi radialis longus (ECRL, extensor carpi ulnaris (ECU), extensor digitorum (ED), Lipoma (*), magnetic resonance imaging (MRI), posterior interosseous nerve (PIN), deep head of supinator (Sd), superficial radial nerve (SRN), superficial head of supinator (Ss)

A 66-year-old gentleman presented with a four-month history of a progressive weakness of finger extension involving all digits of the right hand. On initial clinic evaluation, he had 0 out of 5 strength in the extension of all fingers, including the thumb, but without any weakness of wrist extension. A radial deviation of the wrist was not documented. He did not have any pain or numbness. Electromyography (EMG) and nerve conduction study (NCV) showed posterior interosseous nerve (PIN) entrapment at the arcade of Frohse (AF). Magnetic resonance imaging (MRI) showed a homogeneously hyperintense lesion within the supinator muscle on T1-weighted imaging (Figure 1).

**Figure 2:**
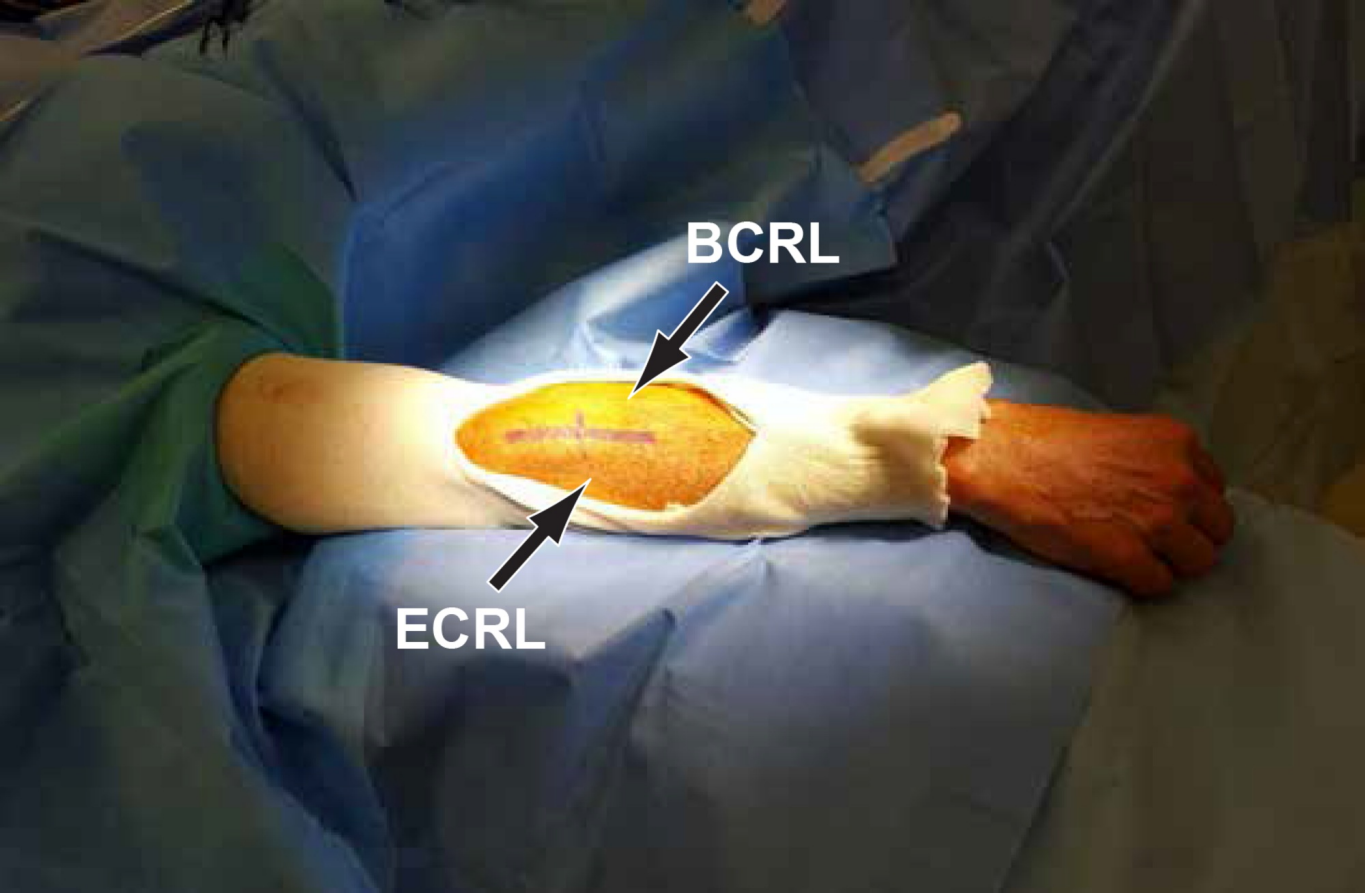
Incision line along the posterior border of the BCRL A 10-centimeter incision line between the brachioradialis (BCRL) and extensor carpi radialis longus (ECRL).

The lesion measured 3 cm medial to lateral, 1.5 cm in depth, and 3.3 cm anterior-posterior. Mass effect was seen on the neurovascular bundle at the AF. A 10-cm incision was made along the posterior border of the brachioradialis (BCRL) muscle (Figure 2).

**Figure 3:**
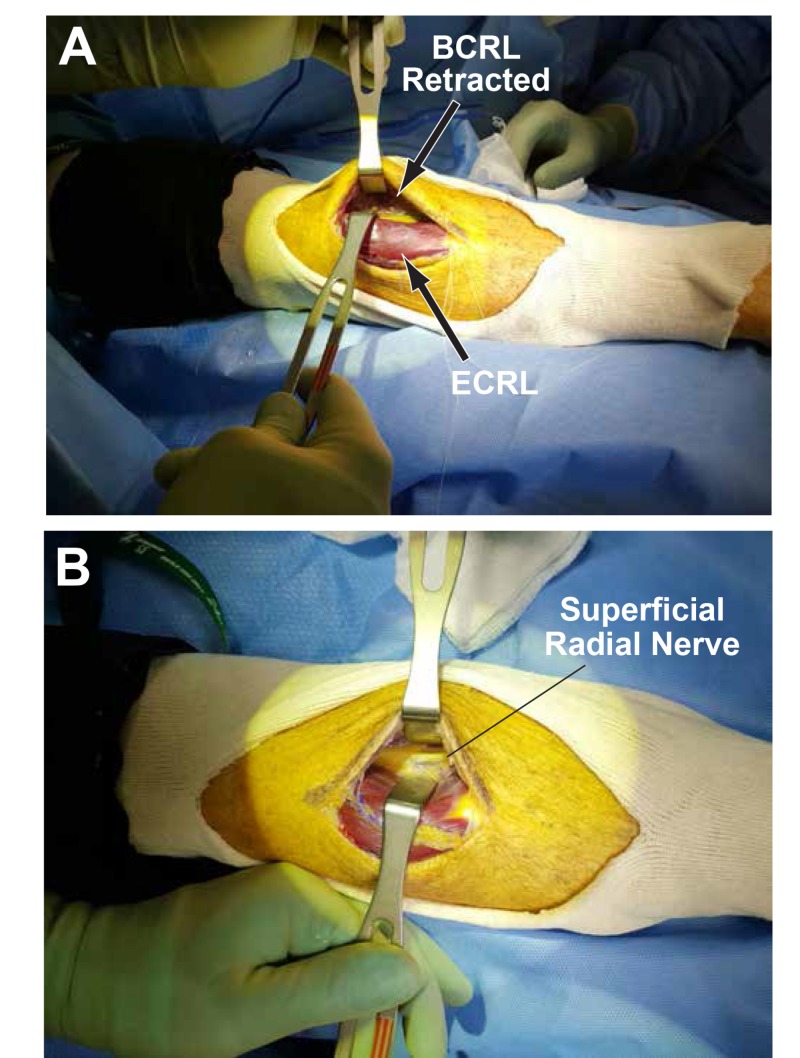
Incised fascia and plane development using blunt dissection (A) The extensor carpi radialis longus (ECRL) and brachioradialis (BCRL) are retracted after developing a plane in between. (B) The BCRL is further retracted to expose the superficial radial nerve.

The fascia in between the brachioradialis (BCRL) and extensor carpi radialis longus (ECRL) was incised and a plane was developed using blunt dissection (Figure 3).

**Figure 4:**
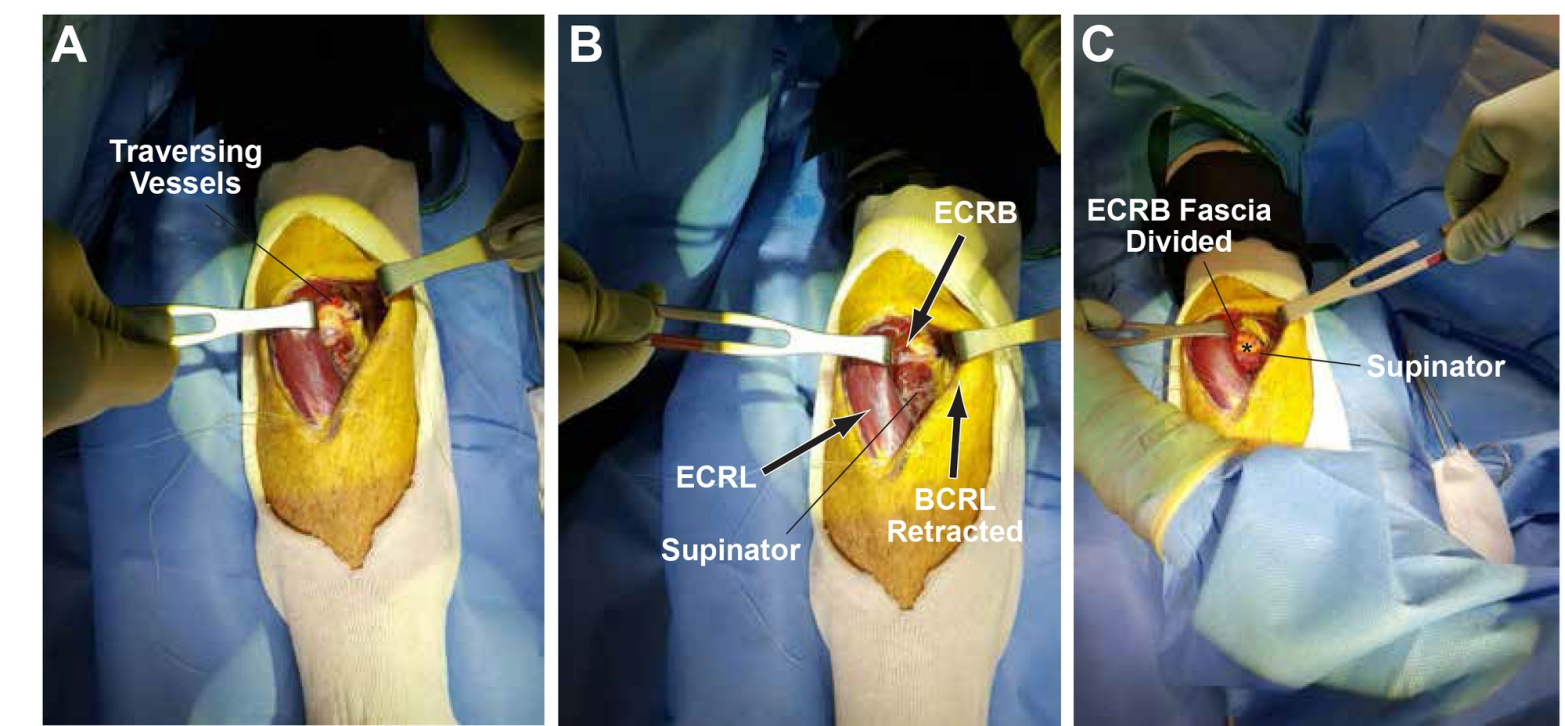
Fascia divided to expose the lipoma (A) Overlying traversing vessels usually present and ligated. (B) The extensor carpi radialis longus (ECRL) and brachioradialis (BCRL) are further retracted to expose the supinator and extensor carpi radialis brevis (ECRB). (C) The extensor carpi radialis brevis (ECRB) fascia divided.

The fascia of the extensor carpi radialis brevis (ECRB) was divided sharply and the lipoma was visible, arising deep to the superficial head of the supinator (Figure 4).

**Figure 5:**
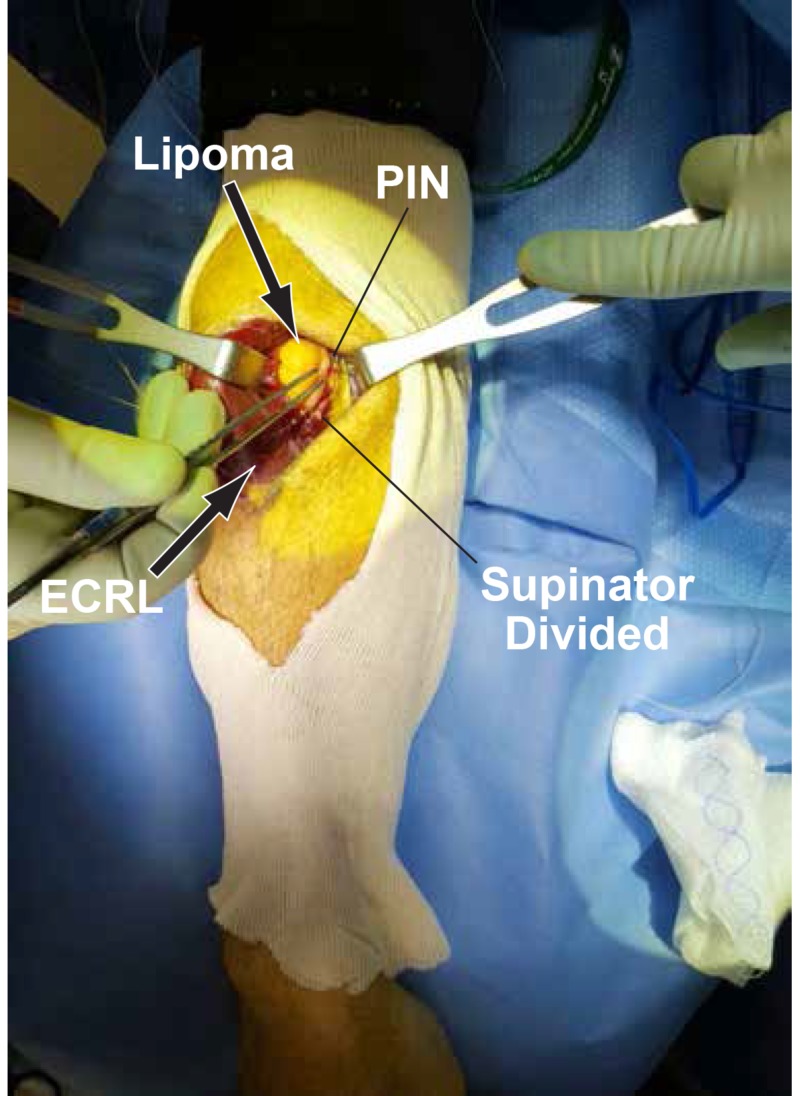
Fully exposed lipoma The arcade of Frohse (AF) and superficial head of supinator are divided to further expose the lipoma. The posterior interosseous nerve (PIN) is also visible.

The radial nerve bifurcation into the superficial radial nerve and PIN was identified. The AF and the superficial head of the supinator muscle were divided until the lipoma was fully exposed (Figure 5).

**Figure 6:**
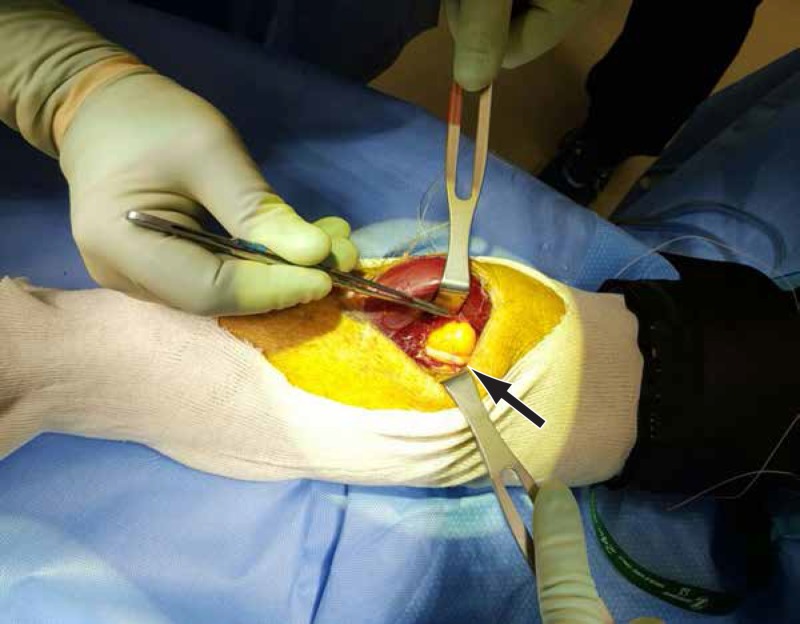
PIN draped across the lipoma The posterior interosseous nerve (PIN) is draped across and attached to the lipoma. There is swelling of the PIN proximal to the constriction site (arrow).

The PIN was draped across the outer surface of the lipoma and then continued its course deep to the remaining superficial head of the supinator (Figure 6).

**Figure 7:**
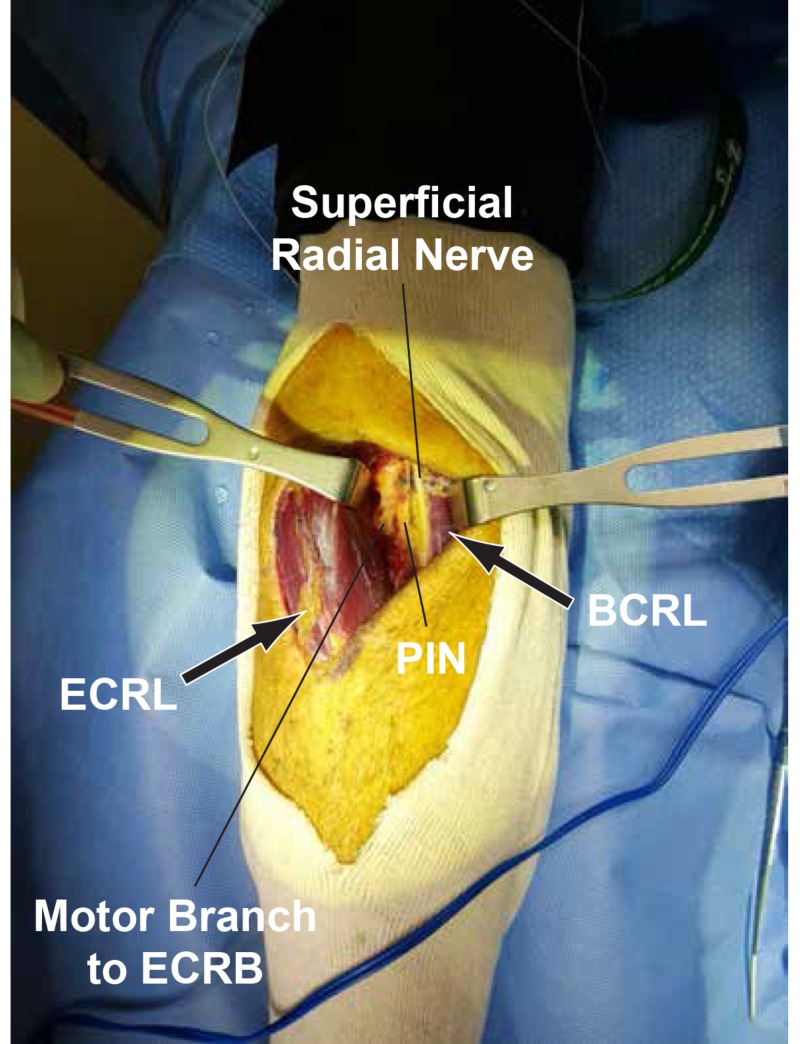
Complete excision of the lipoma After the complete excision of the lipoma from the motor branch to the extensor carpi radialis brevis (ECRB), the posterior interosseous nerve (PIN) and superficial radial nerve are visualized from left to right. The extensor carpi radialis longus (ECRL) and brachioradialis (BCRL) are labeled for reference.

There was a swelling of the PIN proximal to its compression site by the lipoma and the AF. The PIN nerve fibers appeared slightly spread at the attachment site of the lipoma. The PIN was stimulated with a nerve stimulator probe and there was no response distally. We did external neurolysis and swept the PIN laterally to allow for the complete excision of the lipoma (Figure 7).

Pathology revealed mature adipose tissue consistent with lipoma without any nerve fibers. At a four-month follow-up visit, the patient regained 4 out of 5 strength of finger extension in all digits.

## Discussion

Anatomy

After the radial nerve pierces the intermuscular septum, it enters the forearm and bifurcates into the superficial and deep branches. The superficial branch provides sensory innervation to the lateral forearm and the radial half of the dorsum hand. The deep branch of the radial nerve gives direct branches to the BCRL, extensor carpi radialis longus (ECRL), extensor carpi radialis brevis (ECRB), and supinator before continuing in between the superficial and deep layers of the supinator muscle as the PIN [1]. The PIN then exits the supinator and divides into two branches: the descending and recurrent branches [2]. The descending branch provides motor innervation for the abductor pollicis longus, extensor pollicis longus and brevis, and extensor indicis. And the recurrent branch innervates the remaining extensors of the forearm: extensor digitorum communis and quinti and extensor carpi ulnaris (ECU).

Clinical presentation

Symptoms of PIN palsy can be categorized into posterior interosseous syndrome (PIS) or radial tunnel syndrome (RTS) [2]. PIS is classically described as a pure motor weakness without any pain or sensory deficit since the PIN only provides motor innervation to the extensor musculature of the forearm without any cutaneous sensory function and is mainly responsible for the extension of the metacarpophalangeal joints. On the other hand, RTS is characterized by lateral forearm pain without any weakness due to the involvement of the unmyelinated group IV afferent fibers within the PIN carrying nociception from the wrist capsule and forearm musculature [3]. Typically, wrist extension is not significantly affected in PIS since the ECRL and ECRB receive its innervation by the deep branch of the radial nerve proximal to the PIN; however, there may be a radial deviation of the wrist on extension due to ECU weakness.

Extraneural and intraneural lipomas

Lipomas of the peripheral nerves are benign, slow-growing fatty tumors composed of mature white fat cells [4]. Lipomas are most commonly extraneural, causing the compression of the peripheral nerve, although a few case reports of intraneural lipomas, including the PIN, have been described [5-6]. Extraneural lipomas are intramuscular [7], intermuscular [8-9], or parosteal [2,10-11]. Avram and Hynes have also reported a case series of the various PIN lipomas, in which the majority had parosteal origins [10]. Intraneural lipomas have been divided into encapsulated “true intraneural lipomas” and fibrolipomatous hamartoma of the nerve [12]. The former involves a well-encapsulated intraneural lipoma with nerve fibers adherent to the outer capsule wall, whereas the latter consists of fibrous and fatty tissue with nerve fibers within [12]. Previously reported intraneural PIN lipomas are shown in Table 1.

**Table 1:** Previously reported intraneural PIN lipomas PIN: Posterior interosseous nerve

Year	Author	Age	Sex	Symptoms	Lipoma Type	Pathology/Findings	Treatment	Outcome
Current	Patel et al. (current case)	66	M	0/5 finger extension	Intraneural	Mature fatty tissue encapsulated, nerve fibers adherent to outer capsule wall	Gross total resection	4/5 finger extension
2015	Yamamoto et al. [6]	60	F	Asymptomatic	Unclear	Nerve fibers within fatty and fibrous tissue	Subtotal resection	Transient extensor weakness of first and fifth digits
2007	Matsuo et al. [5]	60	M	Numbness dorsal hand, 2~3/5 finger extension	Intraneural	Nerve fibers within fatty tissue	Gross total resection of lipoma and PIN sacrificed, tendon reconstruction	4~5/5 finger extension, 3/5 thumb extension, numbness resolved

Matsuo et al. reported a case in which the PIN could not be preserved since the nerve fibers were significantly spread across the lipoma [5]. The patient required tendon reconstruction with a good long-term outcome of finger extension. Yamamoto et al. reported another case of PIN lipoma in an asymptomatic patient where the pathology and clinical findings revealed a fibrous and fatty mass with nerve fibers passing through the mass [6]. Subtotal resection was done to maintain the integrity of the PIN. The patient had temporary weakness post-operatively in finger extension of the first and fifth digits, which later resolved. Our case revealed an encapsulated intraneural lipoma with the PIN adherent to the outer capsule wall with some spreading and swelling of the nerve fibers. We were able to carefully separate the lipoma from the nerve fibers to allow for its gross total resection.

Pre-operative workup

Pre-operative evaluation should include a detailed neurological exam, imaging studies, and EMG/NCV. Sometimes, the mass is prominent and, on palpation, may cause local pain or radiating pain in a specific dermatomal area depending upon which the nerve is involved. Imaging studies include sonography, computerized tomography (CT), and MRI. A lipoma appears as a hyperechoic mass with smooth borders on sonography [13]. A hypoechoic signal within the nerve suggests swelling from the compression [13]. CT may show hyperdense signal changes in the bone if there is an involvement of the periosteum. MRI provides the highest level of detail and can better delineate the spatial relationship between the lipoma and adjacent vasculature, nerves, and musculoskeletal anatomy. The intraneural lipomas are characterized by homogenous hyperintensity on T1- and T2-weighted images [13]. Fibrolipomatous hamartomas are characterized by serpentine-like hypointense nerve fibers within the hyperintense fatty lesion [12]. If present, fibrovascular strands are hypointense on T1-weighted images while cartilaginous areas are intermediate on T1- and hyperintense on T2-weighted images [2]. Finally, EMG and NCV can be useful to locate abnormal nerve conduction.

Management

Treatment for extraneural lipomas involves complete excision and similarly for well-encapsulated intraneural lipomas except in cases where the nerve fibers may be inseparable without damaging the nerve. Recurrence is hardly seen after complete resection but known to occur in cases of subtotal resection. However, for fibrolipomatous hamartomas, radical resection is not possible, and options include subtotal resection for nerve decompression or neurolysis and neurotomy with tendon reconstruction.

Surgical approaches involve the posterolateral and anterior approaches [14-15]. The posterolateral approach involves going through the extensor compartment of the forearm and dissecting in between the extensor digitorum and extensor carpi radialis brevis to reach the supinator, which is then split to see the PIN. The depth of dissection to get to the PIN via the posterolateral approach is less compared to the anterior approach, but dissecting the lipoma away from the nerve is more challenging since the lipoma may hinder direct visualization of the PIN, which can be very sensitive to manipulation. On the contrary, the anterior approach involves going through the flexor compartment of the forearm and dissecting between the BCRL and brachialis to then identify the recurrent radial vessels near the site of the radial nerve bifurcation. The supinator muscle is split and then usually, the PIN can be mobilized under direct vision off to one side to allow for the resection of the lipoma. Therefore, the most recent literature suggests the anterior approach is most often used and shown to have better outcomes [14-15]. However, we used a lesser-reported approach in the literature described by Hall [16], which is a lateral variation of the traditional posterolateral approach that involves going in between the BCRL and the ECRL. This allowed for both short trajectory and direct visualization of the PIN. In addition, the proximal and distal PIN can be exposed with minimal retraction compared to the anterior approach. Therefore, the anterior approach may not always be the best option, as the literature suggests. Others have described an extensive incision from the lateral aspect of the distal upper arm to the forearm that allows for a combined anterior and posterior approach [17-18].

Surgical resection is usually the treatment of choice in symptomatic cases. If asymptomatic, but the lesion has more aggressive features on imaging, such as enhancement, then surgical treatment should also be considered. The early timing of surgical resection is favored and generally associated with better outcomes when done within seven months of symptom onset. The optimal surgical treatment is debatable in cases of severe PIN compression, chronic compression more than seven months due to a mass lesion, or the absence of nerve action potential. In addition to the removal of the mass lesion, some advocate more aggressive treatment, such as internal neurolysis, neurorrhaphy, nerve grafting, or tendon transfer. Our case is unique in that there was an excellent outcome with just the removal of the mass lesion for decompression despite a severely compressed PIN with swelling and the absence of nerve action potentials distal to the compressions site. Sakamato et al. reported a series of PIN lipomas in which one case presented with complete PIN paralysis and poor recovery despite surgical treatment using the anterior approach; however, the duration of symptoms and intraoperative findings was not mentioned [19]. Interestingly, in that case series, the PIN was pushed anteriorly towards the flexor side by the lipoma in the majority of cases with PIN palsy and pushed more posteriorly towards the extensor side in cases without palsy. Maldanado et al. reported two cases with complete PIN paralysis and symptom durations of eight and 30 months that failed to improve after the resection of the extraneural lipoma and required tendon transfer with favorable outcomes [20]. Despite the complete PIN paralysis in our case, the prompt surgical intervention and limited duration of symptoms likely allowed for excellent recovery and outcome in our case.

## Conclusions

PIN palsy secondary to intraneural lipomas requires early diagnosis and surgical intervention for optimal outcome. The optimal surgical approach can vary depending on the anatomical relation of the lipoma and the neurovascular structures. Our case illustrates a variation of the traditional posterolateral approach for the complete excision of an intraneural lipoma and an excellent outcome despite complete PIN paralysis on presentation.
